# Bacterial Colony: First Report of Donut Colony Morphology among Diphtheroids Isolated in Blood

**DOI:** 10.7759/cureus.374

**Published:** 2015-11-04

**Authors:** Venkataramana Kandi

**Affiliations:** 1 Department of Microbiology, Prathima Institute of Medical Sciences

**Keywords:** donut colony, diptheroids, blood

## Abstract

Isolation of diphtheroids in human clinical specimens is not uncommon. Several studies have highlighted the significance of these bacteria in human infection, which morphologically resemble Corynebacterium diphtheriae. Previous studies have noted that occurrence of these bacteria in specimens like the blood should not be ignored as they can result in serious infections like endocarditis and sepsis among debilitated individuals, including the neonates. We report isolation of diphtheroid bacterium in blood from a case of septicaemia showing donut colony morphology.

## Introduction

A single clone of bacteria multiplying in the presence of nutrients to form a visible growth on the solid medium is called a bacterial colony. Colony morphology is one among the various characters of bacteria, which is also unique to a particular genus of bacteria that could be instrumental in preliminary identification. Size (measured in millimetres - pinpoint (≤ 1 mm), small (2-3 mm), medium (4-5 mm), and large (> 5 mm) colonies), shape (circular, irregular, rhomboid, umbonate, umbonate, filamentous, or rhizoid), odour (curdy, fruity, pungent, buttery, bleachy, putrid), texture (smooth, transparent, opaque, translucent, metallic sheen, glistening, rough, wrinkled, moist, mucoid, pitting, or corroding) elevation over the solid medium (flat, raised, or convex), margins of the colony (entire, filamentous, or irregular), and pigment production (yellow, golden yellow, whitish, green, bluish-green, or orange) are among the most common physiological properties of bacteria, necessitating their identification and characterization [1].

Among the members of the genus Corynebacterium*,* only C diphtheriae is an established pathogen. Other members are present either as normal flora in human or as saprophytes in the environment and have rarely been associated with human infections. Recently, there has been an increased report of both a new species of genus Corynebacterium and their occurrence in various human infections. It assumes significance for clinical microbiologists and clinicians to understand the potential role of diphtheria-like bacteria in human infections [2]. Only a few studies globally have characterized the human clinical isolates of diphtheroids and their antimicrobial susceptibility patterns [3-4]. This communication presents the appearance of a rare colony type of diphtheroids and reemphasizes the possible potential pathogenic nature of non-diphtheritic Corynebacteria and the need for their identification and reporting when isolated from human clinical specimens.

## Materials and methods

### Laboratory methods and rare observation

**Figure 1:**
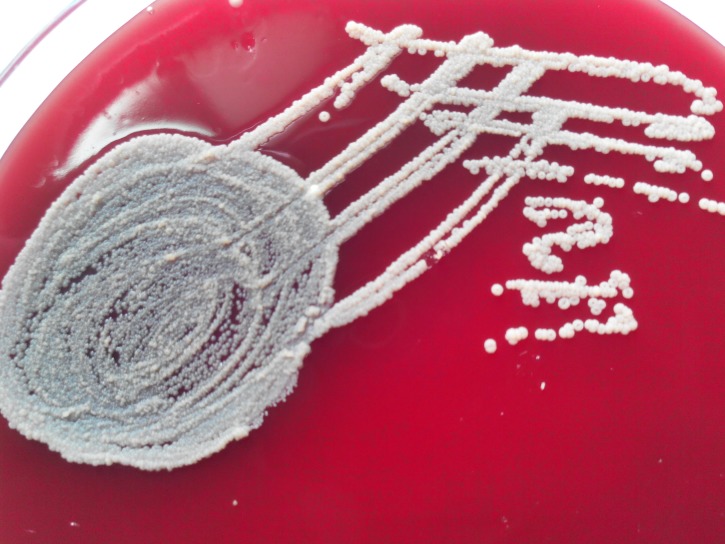
The appearance of donut colony morphology showing raised edges and central halo on blood agar

A very rare colony type showing donut-shaped morphology was observed on blood agar on first isolation. This bacterium was isolated from a case of septicaemia in a blood culture. The growth was noted after two days of incubation in an automated blood culture system. Subculture onto blood agar and MacConkey’s agar under aerobic incubation at 35^0^-37^0^ C revealed 2-3 mm small, round, whitish coloured, donut-shaped colonies on blood agar as shown in Figure 1.

**Figure 2:**
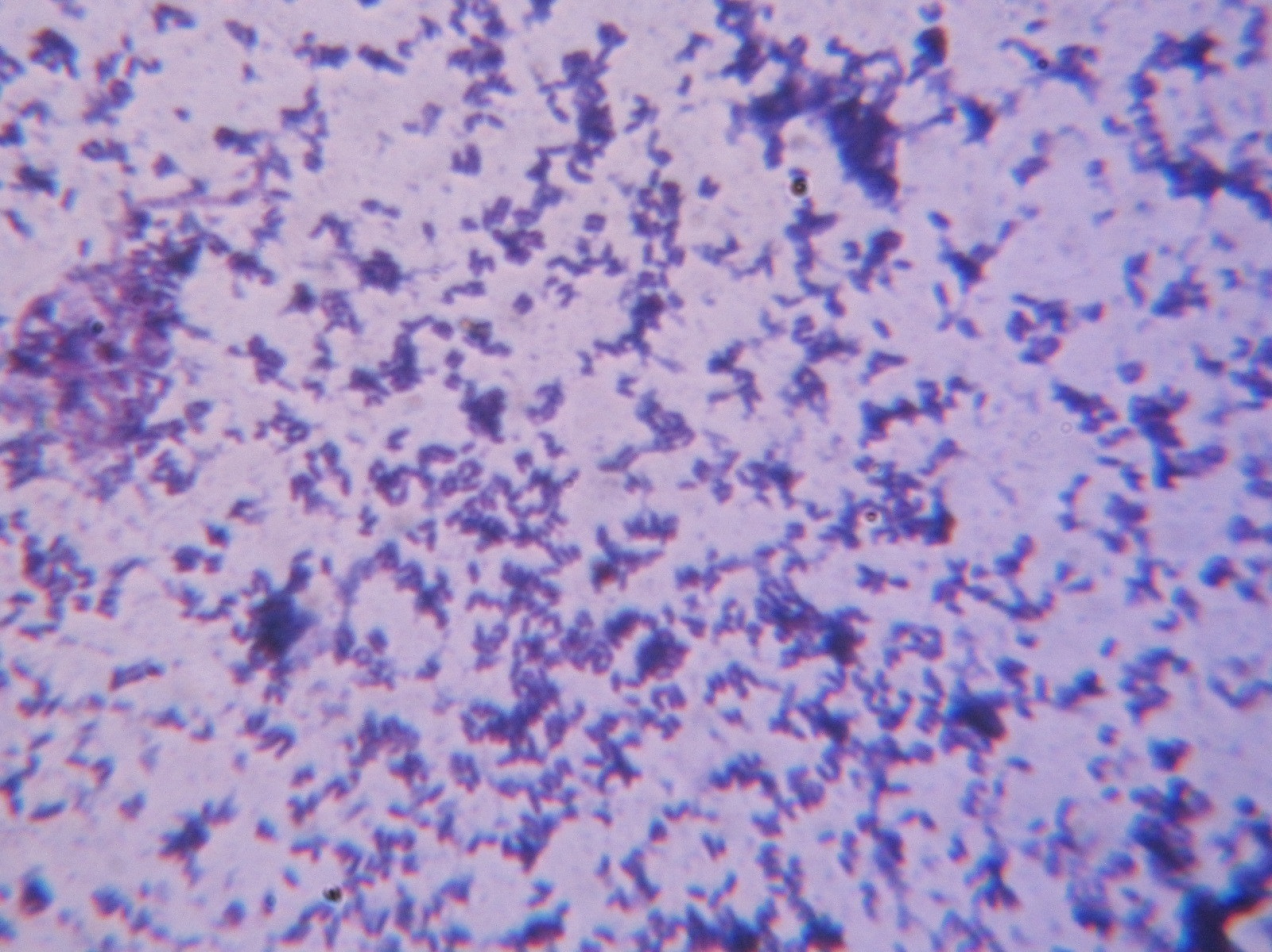
Grams stain showing gram positive bacilli resembling Corynebacterium diphtheriae

There was no growth observed on MacConkey’s agar. The bacteria were found to be not easily emulsifiable, and on gram’s stain, rod-shaped bacteria, slightly curved with a beaded appearance, morphologically resembling Corynebacterium diphtheriae, were observed with variable staining, as some appeared gram-negative and others appeared gram-positive as shown in Figure 2.

Since gram staining was indeterminate, we tried alternate and non-staining methods to determine if the isolated bacterium had a gram-positive or a gram-negative cell wall. KOH test and the antibiotic disc test (vancomycin, 5 µg, and colistin, 10 µg) are the alternates to gram stains wherein gram-positive bacteria are KOH string test negative, vancomycin 5 µg – sensitive and colistin 10 µg - resistant. Gram-negative bacteria are positive for KOH string test, vancomycin 5 µg -resistant and colistin 10 µg – sensitive [5-6].

**Figure 3:**
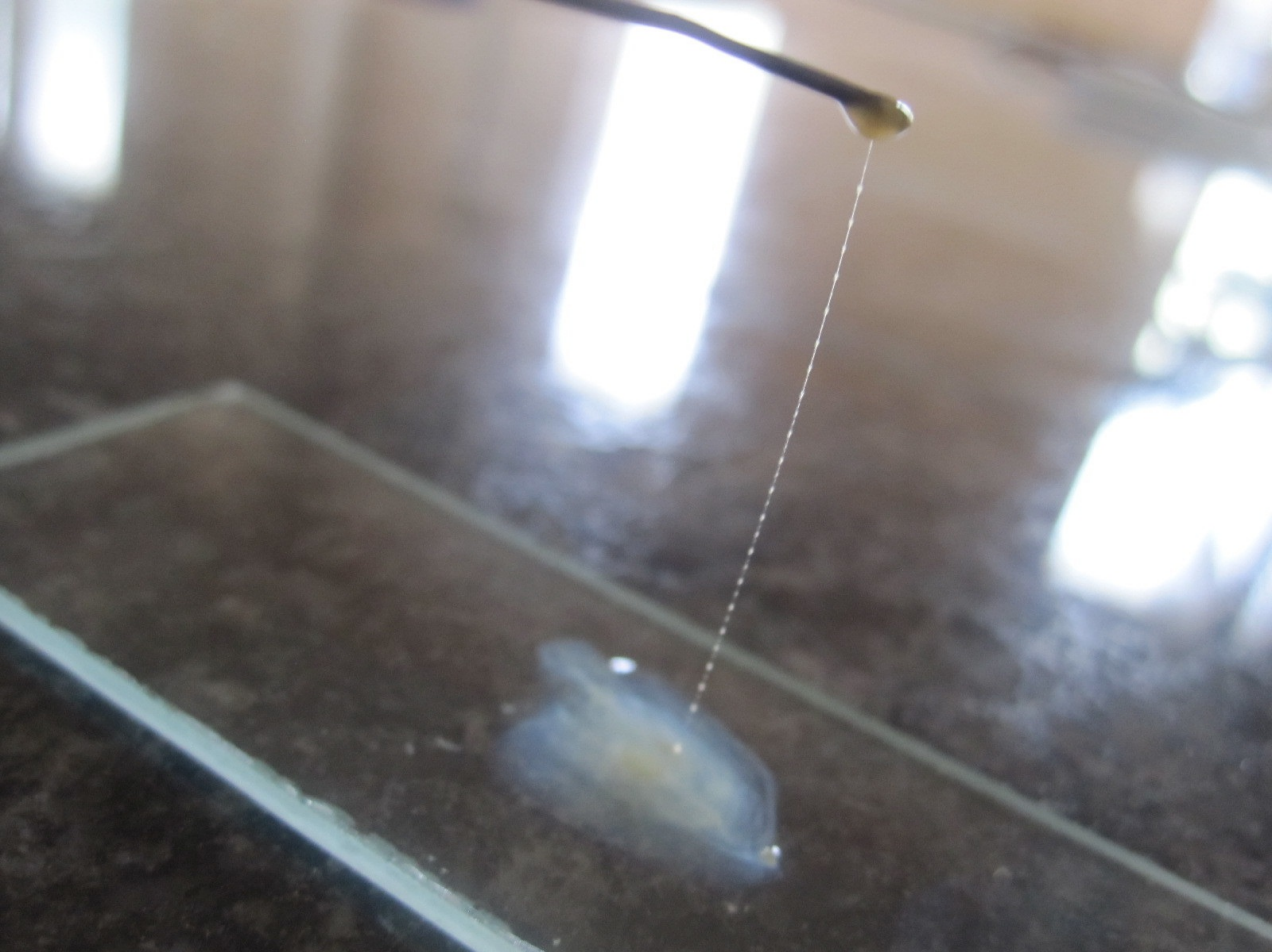
String test for the determination of cell wall (gram-positive/gram-negative)

For the KOH test, the bacterial colony is picked up with the help of an inoculating straight wire and is emulsified in a drop/10 µl of 3% KOH on a clean and grease-free slide. After stirring/mixing the colonies for about 60 seconds, one should look for the appearance of a viscous nature and, in that case, the loop should be raised very slowly to see the formation of stringiness indicating that it is a gram-negative bacterium. Gram-positive bacteria don’t form a viscous emulsion and are negative for string-like formation as shown in Figure 3.

## Results

**Figure 4:**
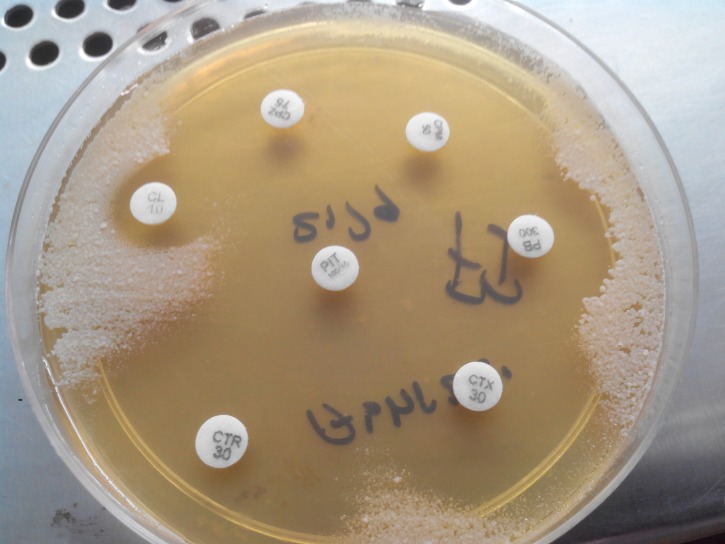
Antimicrobial susceptibility testing plate

The isolated bacterium was found to be negative for string test, was resistant to vancomycin, 5 µg, and sensitive to colistin, 10 µg. Biochemically, the bacterium was observed to be catalase-positive, indole, urease, and citrate-negative, and showed no reaction/inert in triple sugar iron agar (TSI), indicating that the organism has neither the ability to metabolise sugar nor the peptone. Antimicrobial susceptibility pattern of the isolated bacterium revealed sensitivity to imipenem, ciprofloxacin, ofloxacin, amikacin, piperacillin-tazobactam, and cephalosporins, including ceftriaxone, cefotaxime, ceftazidime, cefepime, and cefepime-tazobactam, and resistant to trimethoprim-sulfamethoxazole as shown in Figure 4.

The isolated bacterium was grouped as belonging to diphtheroid, based on conventional methods and was not speciated. The bacterium is being sent for identification by advanced identification systems that include molecular methods.

## Discussion

As evidenced from recent reports, bacteria have the ability to form different colony types. These colonial characters could be species specific, aiding in their preliminary identification, and also demonstrating certain characteristics of the bacterial species, e.g. the mucoid-type colonies that are usually seen among capsule-producing organisms [7-8]. Variations in colony morphology are not uncommon and previous literature has noted that microorganisms exposed to mutagenic agents like ultraviolet rays produce morphologically mutant types showing colony morphologies (donut, walnut, frilly, echinoid) distinct to their natural phenomenon. Molecular studies using pulse-field gel electrophoresis (PFGE) indicated that these microorganisms demonstrated atypical colony attributed to chromosomal rearrangement resulting from mutations [9]. Previous studies have noted the formation of donut shaped colonies among *Candida* spp and fungal plant pathogens (Ensifer medicae strain, Ustilago maydis) and soil fungus (Mortierella spp) [10-11].

Bacteria cells showing donut appearance was previously noted among *Campylobacter* spp under an electron microscope. Although the colony, on the whole, appeared normal, the cells lining the colony surface showed donut shape. This study has concluded that the donut shape of cells is an intermediary morphological form between the young spiral forms and the older coccoid forms [12]. Another previous report has hypothesized that under stress (heat, freezing, radiation, exposure to chlorine and copper) bacteria like Campylobacter spp, Yersinia enterocolitica, which are bacilli, could produce donut cells, and this phenomenon might also be seen among Staphylococcus spp [13]. Streptococcus pneumoniae colonies were also previously reported to produce donut-shaped colonies on blood agar isolated from a throat swab [14]. Bacteria have been noted to respond to the external stimuli/variable environmental conditions that include immune response, physical stress, antibiotic stress, and nutritional deficiencies by acquiring survival strategies in the form of altering cell morphologies (curved/rod-filamentous), biofilm production, small colony variants, and quorum sensing [15-18].

## Conclusions

Considering the fact that bacteria are isolated from clinical specimens by growing them on solid media, microbiology trainees and clinical microbiologists should utilize the colony morphology as a primary tool in the identification of bacteria. Although basic colony morphologies have been described in various standard text books, laboratory specialists should continuously update the changing trends of bacterial colony characters. This appears to be the first time that a colony resembling donut morphology is reported in the literature.
